# CT fatty muscle fraction as a new parameter for muscle quality assessment predicts outcome in venovenous extracorporeal membrane oxygenation

**DOI:** 10.1038/s41598-020-79495-5

**Published:** 2020-12-28

**Authors:** Anton Faron, Stefan Kreyer, Alois M. Sprinkart, Thomas Muders, Stefan F. Ehrentraut, Alexander Isaak, Rolf Fimmers, Claus C. Pieper, Daniel Kuetting, Jens-Christian Schewe, Ulrike Attenberger, Christian Putensen, Julian A. Luetkens

**Affiliations:** 1 Department of Radiology and Quantitative Imaging Lab Bonn (QILaB), University Hospital Bonn, Venusberg-Campus 1, 53127 Bonn, Germany; 2 Department of Anesthesiology and Intensive Care Medicine, University Hospital Bonn, Venusberg-Campus 1, 53127 Bonn, Germany; 3 Department of Medical Biometry, Informatics, and Epidemiology, University Hospital Bonn, Venusberg-Campus 1, 53127 Bonn, Germany

**Keywords:** Prognostic markers, Respiratory distress syndrome, Muscle, Outcomes research

## Abstract

Impaired skeletal muscle quality is a major risk factor for adverse outcomes in acute respiratory failure. However, conventional methods for skeletal muscle assessment are inapplicable in the critical care setting. This study aimed to determine the prognostic value of computed tomography (CT) fatty muscle fraction (FMF) as a biomarker of muscle quality in patients undergoing extracorporeal membrane oxygenation (ECMO). To calculate FMF, paraspinal skeletal muscle area was obtained from clinical CT and separated into areas of fatty and lean muscle based on densitometric thresholds. The cohort was binarized according to median FMF. Patients with high FMF displayed significantly increased 1-year mortality (72.7% versus 55.8%, P = 0.036) on Kaplan–Meier analysis. A multivariable logistic regression model was built to test the impact of FMF on outcome. FMF was identified as a significant predictor of 1-year mortality (hazard ratio per percent FMF, 1.017 [95% confidence interval, 1.002–1.033]; P = 0.031), independent of anthropometric characteristics, Charlson Comorbidity Index, Simplified Acute Physiology Score, Respiratory Extracorporeal Membrane Oxygenation Survival Prediction Score, and duration of ECMO support. To conclude, FMF predicted 1-year mortality independently of established clinical prognosticators in ECMO patients and may have the potential to become a new muscle quality imaging biomarker, which is available from clinical CT.

## Introduction

In patients with severe acute respiratory failure (ARF), extracorporeal membrane oxygenation (ECMO) may be considered as a treatment option^1^. ECMO was reported to achieve remarkable success for instance in influenza A (H1N1) related ARF^2^ as well as within a large randomized controlled trial^3^. Consequently, the usage of ECMO for treatment of ARF is increasing worldwide^4^. Advances in critical care medicine, including progress in the field of ECMO, have led to improved survival rates in these patients^1,5,6^. However, due its ongoing high incidence, ARF is still considered a major health issue^5,7^. Apart from that, ARF survivors were frequently observed to suffer from persistent sequelae such as physical impairment and reduced mobility, leading to dismal outcomes^8,9^. To some extent, this may be explained by the fact that intensive care unit (ICU) acquired muscle weakness was shown to be frequent among those patients and is related to increased morbidity and mortality on long-term follow up^10,11^. Consequently, the skeletal muscle system is considered a major target and primary determinant of functional recovery and thereby outcome in these patients. Among factors such as age, nutritional status, comorbidities, and administered medications, the baseline muscle functional status is considered to be of vital importance for long-term outcome in ARF^12^. However, as patients with ARF are typically admitted as emergencies, the baseline muscle status is almost always unknown.

Conventional methods for assessment of skeletal muscle function such as measurement of manual grip strength or electromyography^13,14^ require patient cooperation. Therefore, these tests may be considered impractical for skeletal muscle assessment in the ECMO setting. Also, laboratory markers such as albumin^15^ may be limited to assess skeletal muscles in ECMO patients, as laboratory values may be distorted by concomitant disorders or alterations in hydration status. Ultrasound may be used as a bedside imaging modality for skeletal muscle assessment in critically ill patients^16–18^. However, it requires a dedicated examination and measurements may be affected by examiner-related factors such as transducer positioning. Although factors such as changes in hemodynamics complicate conduction of CT in ECMO patients, several clinical scenarios exist which make CT examinations crucial in these patients^19,20^. Skeletal muscles may be assessed from CT for determination of body composition^13^. For instance, in a recent report fatty muscle fraction (FMF), opportunistically obtained from pre-interventional CT, was proposed as an objective measure of muscle quality in patients receiving endovascular aortic valve replacement^21^. Therefore, in this study we aimed to explore whether clinical CT examinations in ECMO may also be exploited for determination of body composition and if the derived measurements may also be related to outcome in these patients.

## Methods

### Study population

**Figure 1:**
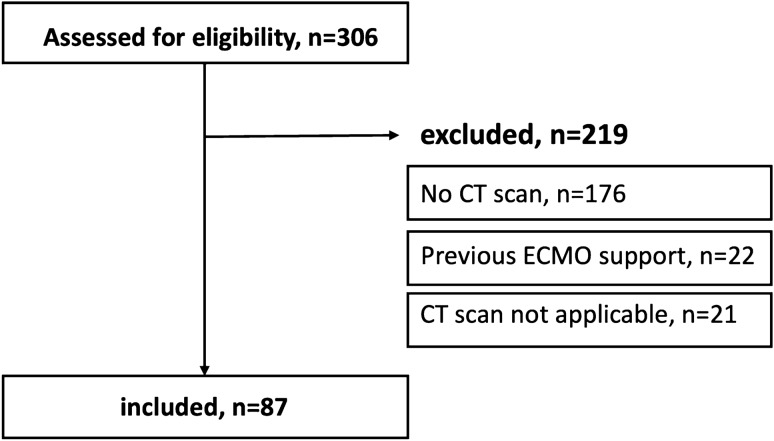
Study inclusion flowchart. Patients who received venovenous ECMO for severe acute respiratory failure between December 2014 and August 2018 at our center were screened for eligibility (n = 306). Patients were excluded if no CT scan within 4 days of ECMO initiation was available (n = 176), if they received previous ECMO support (n = 22), or if CT scans were not applicable (n = 21). *CT* Computed tomography, *ECMO* Extracorporeal Membrane Oxygenation.

Patients who received vv-ECMO for ARF between December 2014 and August 2018 at our tertiary academic center were retrospectively evaluated (Fig. 1). Patients who had diagnostic CT scans within 4 days of initiation of ECMO support were identified. In these patients, the superior mesenteric artery needed to be covered by the field-of-view, as this level served as the anatomical landmark for conduction of muscle measurements. Medical records were reviewed to retrieve clinical variables and baseline physical characteristics of included patients.

### Image analysis

**Figure 2:**
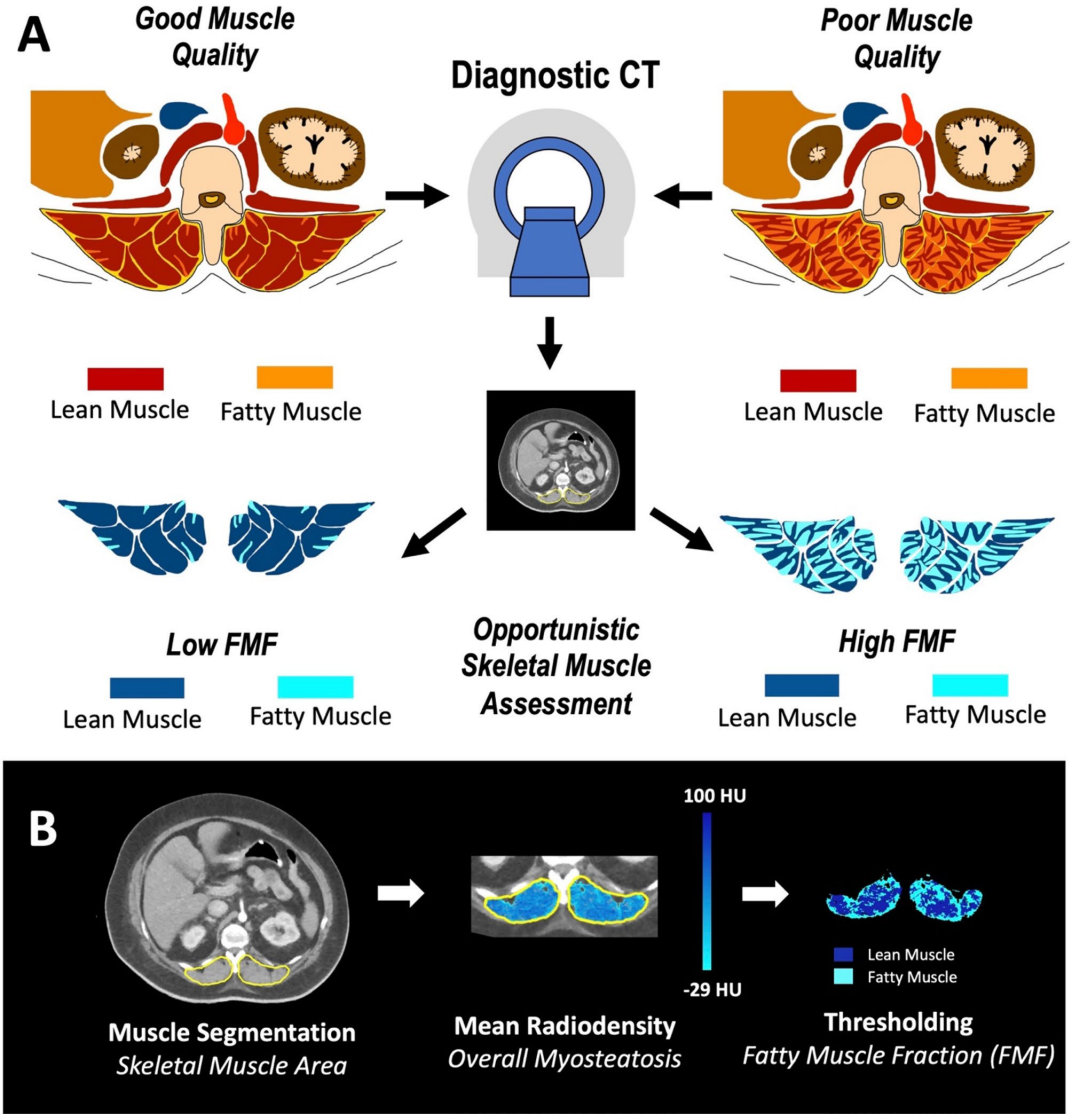
The concept of FMF. (**A**) Skeletal muscle fat infiltration is considered an indicator of muscle quality. Based on densitometric thresholds and accepted cut-off values of lean and fatty skeletal muscle, muscle compartments may be separated into areas of fatty and lean muscle. The FMF is calculated as the area of fatty muscle tissue related to the total skeletal muscle area, resulting in a relative and comparable measure of muscle quality. This biomarker can be opportunistically obtained from clinical CT scans. (**B**) Skeletal muscle area was obtained as the bilateral compartment area of paraspinal skeletal muscles at the level of the superior mesenteric artery. Within the skeletal muscle area, muscle tissue is identified by an attenuation threshold range of 100 to − 29 HU. Mean Radiodensity was highlighted to visualize overall muscle fat infiltration (myosteatosis). Based on thresholds ranges of − 29 to 29 HU for fatty muscle and 30 to 100 HU for lean muscle, FMF was calculated. *CT* Computed tomography, *FMF* Fatty muscle fraction, *HU* Hounsfield units.

Skeletal muscle measurements were performed at the level of the origin of the superior mesenteric artery, as this level is often covered also in chest CT protocols. For each patient, a single axial cross-sectional image at the root of the superior mesenteric artery was retrieved. Tissue areas at this level were previously demonstrated to be highly correlated with total compartment volumes and corresponding measurements were shown to be highly reproducible^22,23^. CT exams were exported from the institutional picture archiving and communication system (IMPAX, Agfa Healthcare, Belgium), de-identified, and transferred to a conventional radiological workstation for image analysis. Image analysis was performed using an in-house software written in MATLAB (Mathworks, Natick, MA, USA) with details of the software reported elsewhere^23^. All muscle measurements were performed by one radiologist (AF) with 3 years of experience in body composition analysis, who was blinded to patient data, not involved in patient treatment and unaware of outcome parameters. For segmentation of the total skeletal muscle compartment, the thoracolumbar fascia was carefully traced bilaterally, separating both the left and right paraspinal skeletal muscle compartment from adjacent tissues^24,25^. Within the total skeletal muscle compartment, skeletal muscle tissue was identified by an attenuation threshold range − 29 to 100 HU^26,27^. For calculation of paraspinal skeletal muscle area, pixels within the defined attenuation threshold range were counted up and multiplied with the respective pixel surface area. Next, to quantify skeletal muscle fat infiltration, the paraspinal skeletal muscle area was separated into areas of fatty and lean muscle based on commonly accepted attenuation thresholds^26^. Accordingly, fatty and lean muscle were defined by attenuation threshold ranges of low (− 29 to 29 HU) and normal muscle attenuation (30 to 100 HU), respectively. Fatty Muscle Fraction (FMF) was then calculated as the area of low attenuation muscle tissue referred to the skeletal muscle area^21^ (Fig. 2).

### Statistical analysis

SPSS Statistics 24 (IBM, Armonk, NY, USA) and Prism 8 (GraphPad Software, La Jolla, CA, USA) were used for statistical analysis. Data ware checked for normal distribution using the Shapiro–Wilk test. If normally distributed, continuous variables are expressed as means with standard deviation and otherwise are given as medians with 25th and 75th interquartile ranges (IQR). Categorical data are presented as absolute frequencies. For survival analysis, the cohort was binarized based on median FMF with a cutoff value of > 44% to define high FMF. The Kaplan–Meier method with log-rank tests was used to compare survival between these two groups. For group comparison of continuous variables, unpaired *t*-test and Mann Whitney *U* test were used for parametric and nonparametric testing, respectively. Categorical data were compared using either χ^2^ test or Fisher’s exact test, as appropriate. A multivariable logistic regression model was built to determine the impact of FMF and clinical variables on 1-year mortality. Covariates were selected a priori, including FMF as a continuous variable alongside with a set of routinely available factors that were judged to have a potential impact on outcome based on clinical judgement. These factors were the Respiratory ECMO Survival Prediction (RESP) score as it was previously proposed as a validated measure to predict survival in ECMO patients^1^, the Charlson Comorbidity Index (CCI) as an indicator of severe comorbidities, the Simplified Acute Physiology Score II (SAPS II) as an indicator of disease severity, the duration of ECMO support as well as baseline anthropometric characteristics such as the body mass index (BMI). Results of the cox regression analysis are displayed as adjusted hazard ratio (HR) with 95% confidence interval (CI). Spearman correlation coefficient was calculated to test the correlation between FMF and continuous clinical factors within the model. The level of statistical significance was set to P < 0.05.

### Ethical approval and informed consent

The study was approved by the institutional review board of the University of Bonn (Institutional Review Board registry number, 283/18) and the need for written informed consent was waived due to the retrospective monocentric study design. The study was carried out in compliance with the ethical standard set in the 1964 Declaration of Helsinki as well as its later amendments.

## Results

### Study population

A total of 87 patients (58 male) with a mean age 54 ± 13 years and applicable CT scans (median ∆_CT to ECMO_, 0; IQR, 0 to 1 days) were identified. Median duration of vv-ECMO support was 11 days (IQR, 7 to 17 days) and the majority of patients received ECMO therapy due to pulmonary acute respiratory distress syndrome (53/87, 61%) while the remainder patients suffered from extrapulmonary acute respiratory distress syndrome (34/87, 39%). At time of ECMO implantation, patients had a median Charlson Comorbidity Index (CCI) of 2 (IQR, 1 to 3). Mean Simplified Acute Physiology Score (SAPS II) 24 h after intensive care unit (ICU) admission was 45.2 ± 12.9. The median predicted survival probability according to Respiratory ECMO Survival Prediction (RESP) score was 45% (IQR, 39 to 65%). In 50/87 patients (58%), weaning from ECMO was successful. One-year survival after initiation of ECMO-support was 31/87 (36%).

### Body composition assessment

Patients had a median body mass index (BMI) of 28.6 kg/m^2^ (IQR, 24.7 to 34.7 kg/m^2^). According to the World Health Organization (WHO) definition of obesity, 61/87 (70%) and 36/87 (41%) were categorized as overweight (BMI, ≥ 25 kg/m^2^) and obese (BMI, ≥ 30 kg/m^2^), respectively. Mean bilateral total skeletal muscle compartment area and consecutive skeletal muscle area were 49.7 ± 14.2 cm^2^ and 44.1 ± 11.8 cm^2^, respectively. Mean skeletal muscle area corrected for body height (skeletal muscle index) was 25.2 ± 6.1 cm^2^/m^2^. Mean radiation attenuation of the skeletal muscle area was 26.9 ± 12.0 HU. For calculation of FMF, the entire respective skeletal muscle area for each patient was subdivided into areas of fatty and lean muscle according to radiation attenuation thresholds. Median FMF for the entire study population was 44% (IQR, 29 to 65%).

### Fatty muscle fraction and clinical outcome

The study cohort was binarized according to median FMF into patients with high (N = 44) and low FMF (N = 43). Patients with high FMF were significantly older (57.3 ± 12.3 years versus 50.3 ± 13.7 years, P = 0.014), had higher BMI (30.9 kg/m^2^ versus 26.6 kg/m^2^, P = 0.018), and were more likely to be female (43% versus 23%, P = 0.049). Regarding clinical characteristics, patients with high FMF had a higher rate of renal failure prior to ECMO therapy (62% versus 29%, P = 0.003), had a significantly lower probability of ECMO survival according to established clinical risk stratification (median RESP score, 45% versus 55%, P = 0.011), and were less likely to undergo successful weaning from ECMO support (46% versus 70%, P = 0.022) (Table 1). Regarding muscle measurements, no significant differences with respect to total skeletal muscle compartment area (51.3 ± 14.8 cm^2^ versus 48.0 ± 13.4 cm^2^, P = 0.275), skeletal muscle area (43.3 ± 11.3 versus 44.9 ± 12.4 cm^2^, P = 0.530) as well as skeletal muscle index (25.0 ± 5.9 cm^2^/m^2^ versus 25.5 ± 6.4 cm^2^/m^2^, P = 0.758) were observed.

**Table 1:** Clinical characteristics of the study population.

Variable		High FMF (N = 44)	Low FMF (N = 43)	P value	Test value
Age (years)		57 ± 12	50 ± 14	0.014	t = 2.498
Sex (male)		25 (57%)	33 (77%)	0.049	χ^2^ = 3.886
Body Mass Index (kg/m^2^)		30.9 (25.7–38.1)	26.6 (23.7–31.8)	0.018	U = 619
Total skeletal muscle compartment area (cm^2^)		51.3 ± 14.8	48.0 ± 13.4	0.275	t = 1.098
Skeletal muscle area (cm^2^)		43.3 ± 11.3	44.9 ± 12.4	0.530	t = 0.630
Skeletal muscle index (cm^2^/m^2^)		25.0 ± 5.9	25.5 ± 6.4	0.758	t = 0.309
Muscle mean attenuation (HU)		17.9 ± 8.2	36.1 ± 7.5	< 0.001	U = 40
Pre-ECMO cardiopulmonary resuscitation		5 (11%)	9 (21%)	0.206	χ^2^ = 1.597
Pre-ECMO renal failure (CRRT)		24 (62%)	12 (29%)	0.003	χ^2^ = 8.901
Charlson comorbidity index		2 (1–3)	2 (1–3)	0.798	U = 916
Simplified acute physiology score II		47.0 ± 12.8	43.2 ± 13.0	0.208	t = 1.269
Diagnosis (pulmonary acute respiratory distress syndrome)		27 (61%)	26 (61%)	0.931	χ^2^ = 0.007
Days on mechanical ventilation prior initiation of ECMO support	< 2	20 (46%)	26 (60%)	0.161	χ^2^ = 1.966
	2–7	16 (36%)	13 (30%)	0.544	χ^2^ = 0.368
	> 7	8 (18%)	4 (9%)	0.230	χ^2^ = 1.442
Respiratory ECMO survival prediction (RESP) score (%)		45 (35–50)	55 (40–75)	0.011	U = 633
Duration of ECMO support (days)		12.5 (8.0–18.0)	9.0 (7.0–16.0)	0.266	U = 814.5
Weaning from ECMO		20 (46%)	30 (70%)	0.022	χ^2^ = 5.259

The cohort was binarized into patients with high and low FMF with a cutoff value of > 44% to define high FMF. Continuous variables are expressed as means with standard deviation while categorical variables are given as total counts and percentages. *CRRT* Continuous Renal Replacement Therapy, *ECMO* Extracorporal Membrane Oxygenation, *FMF* Fatty muscle fraction, *RESP* Respiratory ECMO Survival Prediction Score.

**Figure 3:**
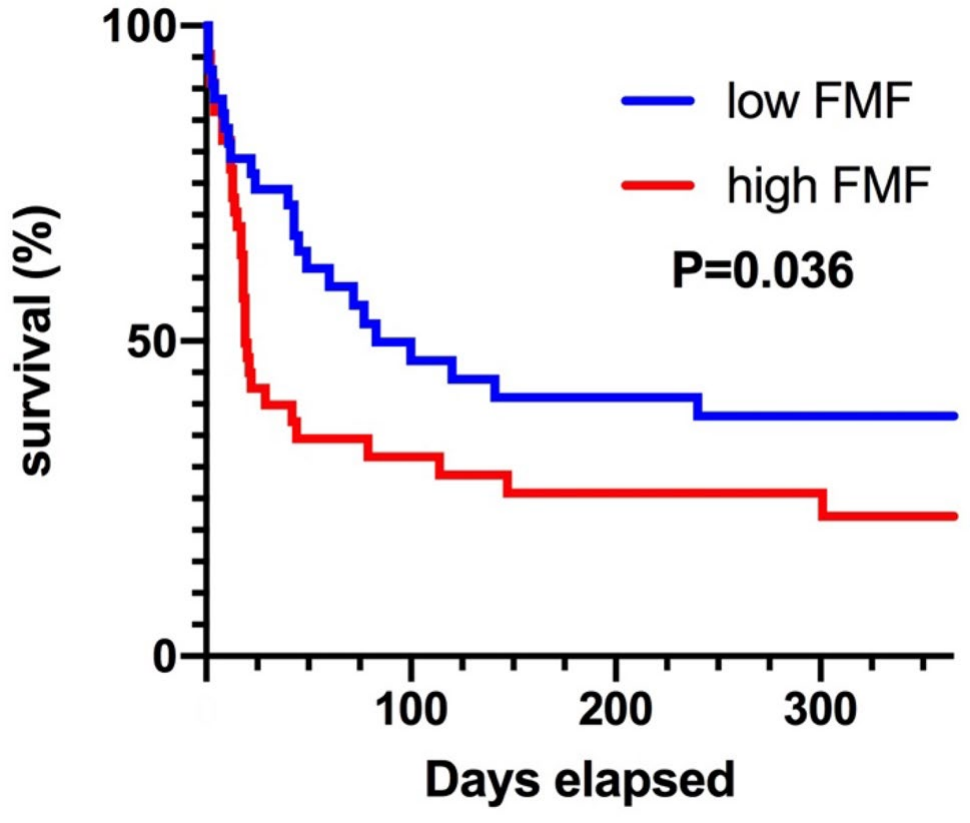
Survival curve. Kaplan–Meier curve illustrating 1-year mortality of patients with high FMF compared to patients with low FMF. The entire study population (n = 87) was binarized based on median FMF with a cutoff value of > 44% to define high FMF. Patients with high FMF displayed significantly increased 1-year mortality following ECMO-implantation for acute respiratory failure (72.7% versus 55.8%, P = 0.036). *ECMO* Extracorporeal Membrane Oxygenation, *FMF* Fatty muscle fraction.

**Figure 4:**
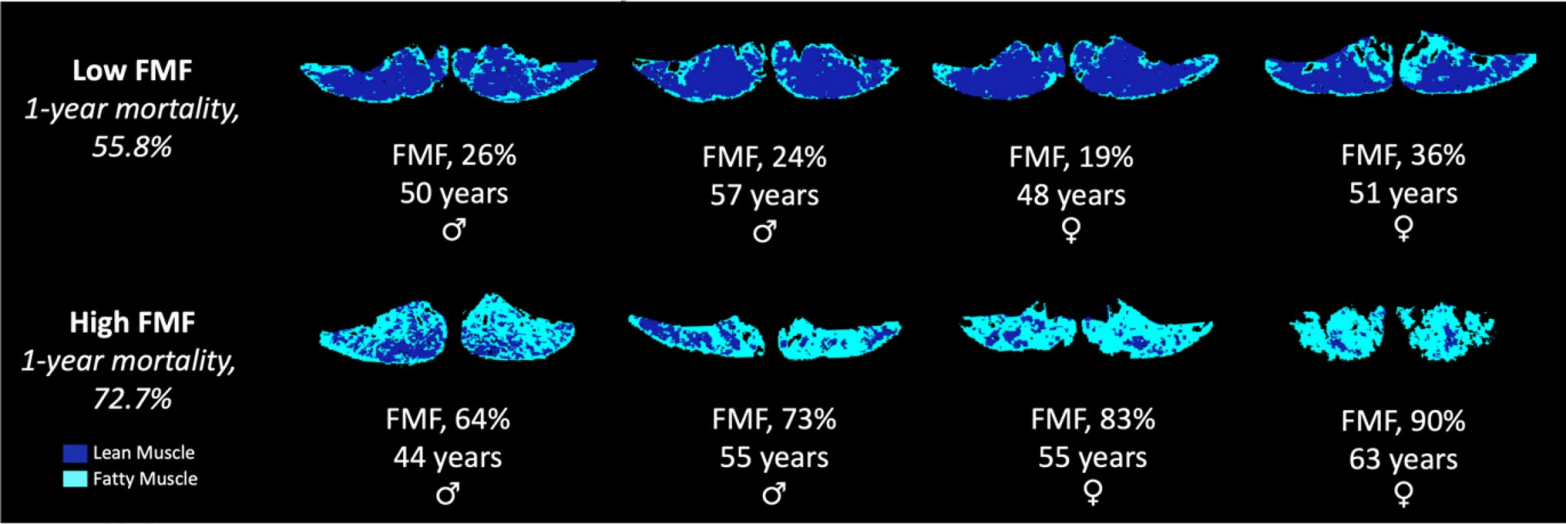
Exemplary patients. The paraspinal skeletal muscle area at the level of the superior mesenteric artery was separated into areas of fatty and lean muscle based on attenuation threshold ranges from CT scans around 4 days of ECMO implantation for acute respiratory failure. FMF was calculated at the fatty muscle area related to the total skeletal muscle area. Based on median FMF of the entire study population (n = 87), patients were binarized with a cutoff value of > 44% to define high FMF. Panels show exemplary patients with high and low FMF with corresponding 1-year mortality rates. *CT* computed tomography, *ECMO* Extracorporeal membrane oxygenation, *FMF* Fatty muscle fraction.

However, patients with high FMF displayed significantly decreased mean radiation attenuation of the skeletal muscle compartment compared to patients with low FMF (mean radiation attenuation of skeletal muscle area, 17.9 ± 8.2 HU versus 36.1 ± 7.5 HU, P > 0.001). According to Kaplan–Meier analysis, high FMF was associated with a significantly increased 1-year mortality following ECMO-implantation (72.7% versus 55.8%, P = 0.036) (Fig. 3). Exemplary images with high and low FMF are provided in Fig. 4. On multivariable analysis, FMF was identified as a risk factor for 1-year mortality following ECMO (hazard ratio per percent FMF, 1.017 [95% confidence interval, 1.002–1.033]; P = 0.031), independent of anthropometric parameters (age, P = 0.430; sex, P = 0.358; BMI, P = 0.339), duration of ECMO support (P = 0.583), severe systemic comorbidities (CCI, P = 0.732), severity of disease (SAPS II, P = 0.570), and clinical risk stratification (RESP score, P = 0.080) (Table 2). No correlation between FMF and continuous clinical factors within the model was observed (age, P = 0.496; BMI, P = 0.569; CCI, P = 0.490; RESP Score, P = 0.232; duration of ECMO support, P = 0.152; SAPS II, P = 0.929).

**Table 2:** Multivariable risk factor analysis for 1-year mortality.

Variable	P value	Hazard ratio (95% CI)
Age	0.430	1.013 (0.981–1.047)
Sex	0.358	1.388 (0.690–2.791)
Body Mass Index	0.339	0.984 (0.952–1.017)
Charlson comorbidity index	0.732	0.975 (0.845–1.126)
Respiratory ECMO survival prediction score (%)	0.080	0.979 (0.955–1.033)
Duration of ECMO support	0.583	1.007 (0.982–1.033)
Simplified acute physiology score II	0.570	1.010 (0.977–1.043)
FMF percent	0.031	1.017 (1.002–1.033)

A multivariable logistic regression model was built to test the impact of clinical variables on 1-year mortality. FMF was included as a continuous variable alongside with a set of a priori selected covariates, which were judged reasonable based on previous literature and clinical perception. Results are displayed as adjusted hazard ratio (HR) with 95% confidence interval (CI). The level of statistical significance was set to P < 0.05. In this analysis, FMF was identified as a risk factor for 1-year mortality following ECMO, independent of anthropometric parameters (age, sex, BMI), duration of ECMO support, severe systemic comorbidities (Charlson comorbidity index), severity of disease (Simplified Acute Physiology Score II), and clinical risk stratification (Respiratory ECMO Survival Prediction Score). *ECMO* Extracorporal Membrane Oxygenation, *FMF* Fatty Muscle Fraction.

## Discussion

This study investigated the relation of CT-derived FMF as an indicator of muscle quality to outcome in patients receiving vv-ECMO for treatment of acute respiratory distress syndrome. FMF, opportunistically obtained from CT scans at ECMO initiation, was identified as a predictor of 1-year mortality, independent from disease severity, clinical prognosticators, anthropometric characteristics, and comorbidities. FMF as an imaging-based indicator of muscle quality may provide additional information on outcome independent from several clinical factors which are routinely available at time of ECMO implantation. Therefore, determination of FMF may have the potential to improve risk stratification in these patients.

Previous studies investigated ultrasound as a bedside imaging modality for skeletal muscle assessment^16–18^. These studies demonstrated its feasibility to assess skeletal muscles in the ICU setting and showed that muscle dimensions as a surrogate of muscle mass may rapidly decline within a few days of critical illness^16^ and may be related to clinical outcomes, including length of hospital stay and status at ICU discharge^17,18^. In general, ultrasound is therefore considered a reliable instrument for assessment of skeletal muscles^13,28,29^ and is applicable in the ICU setting. However, it requires a dedicated examination and it is possible that its accuracy may be affected by individual factors such transducer positioning and compression as well as muscle contraction.

Due to the limitations with ultrasound and the likelihood of delayed timing with muscle strength testing, CT represents an advantageous imaging modality, providing the opportunity to examine muscle quality from clinically available images. In accordance with international guidelines, CT and MRI as cross-sectional imaging methods may be used to evaluate skeletal muscles^13,30^. In the ECMO setting, particularly CT may be considered attractive as it has a strong role in clinical management, and hence may be exploited for opportunistic muscle assessment^19^. Previous studies have shown that single-slice measurements at several anatomical landmarks may provide accurate estimates of skeletal muscle mass^23,28,31^. To provide the greatest possible clinical usefulness, we decided to perform measurements at the superior mesenteric artery level in this study. Unlike the more common third lumbar vertebra level^13,28^, this anatomical landmark is captured within most abdominal and chest CT imaging protocols, which may increase the number of includable cases, and is easy to identify from the transversal plane. Thereby, we consciously focused on measurement of the paraspinal skeletal muscle compartment, as psoas and abdominal wall muscles typically are not sufficiently expressed at this level.

The observation that age- or disease-related changes in muscle function are frequently not reflected by alterations in muscle dimensions has enforced the interest in assessment of muscle quality^13,28,30^. Excess lipid accumulation within the skeletal muscle compartment is considered a pathological phenomenon indicating lowered muscle quality and is related to poor muscle activity and function^32,33^. CT allows for determination of skeletal muscle fat infiltration based on radiation attenuation^26^. Thereby, fat within the skeletal muscle compartment may be stored as either intermuscular adipose tissue or in form of intramyocellular lipid deposits^33^. Technically it is easy to separate intermuscular fat from skeletal muscles based on attenuation threshold ranges. However, the amount of intramyocellular fat deposits will be missed with this approach.

To account for this, in previous studies skeletal muscle fat infiltration was quantified as the mean radiation attenuation of the entire skeletal muscle compartment^32,34–36^. However, given the fact that accepted cutoff values for definition of fatty (reflecting muscle tissue with excessive intramyocellular fat deposition) and lean muscle exist^26^, this approach may be considered a rather vague measurement. Contrary to that, the concept of FMF is to provide an intuitive and comprehensible marker for profound characterization of skeletal muscle quality, irrespective of surrounding tissues. Moreover, FMF with its relative unit does not require further normalization and is independent from anthropometric characteristics^21^.

In accordance with literature, patients in our study presenting lowered muscle quality were older, more likely to be female, and had higher BMI values^34^. These patients had a lower probability of survival, indicated by lowered RESP score, were less likely to undergo successful weaning from ECMO, and displayed increased 1-year mortality. Taken together, these patients were less likely to experience beneficial long-term outcome. Also, we observed a higher rate of patients requiring continuous renal replacement therapy due to renal failure in the lowered muscle quality group. This finding is in line with a recent report, which observed decreased skeletal muscle radiation attenuation in patients with renal failure compared to healthy subjects, indicating a potential association between altered renal function and skeletal muscle fat infiltration^37^. However, as this was not the primary focus of our study, future investigations are needed to further elucidate this relationship.

FMF as a measure of baseline muscle quality was associated with 1-year mortality independent from several clinical factors in our study, including severity of disease as reflected by SAPS II score. Notably, in this study we did not observe a correlation between the degree of skeletal muscle fat infiltration at time of ECMO initiation, as reflected by FMF, and severity of disease. Muscle weakness in ARDS may be subdivided into an early and late phase, with the early phase mainly driven by systemic inflammation due to the underlying disease and the late phase caused by continued muscle disuse after inflammation resolved^12^. Having this concept in mind, it is conceivable that the severity of illness may contribute to muscle wasting during ICU stay. A longitudinal and particularly prospective study setting may be suitable to further address this issue. Also, within these studies potential longitudinal changes in FMF during course of disease may be further elucidated.

We acknowledge some limitations of our study. First, due to the retrospective study design we were unable to determine whether the observed survival disadvantage of poor muscle quality was also related to lowered functional recovery. However, this was not our primary goal. Rather, we propose an easy and clinically applicable alternative method to obtain a surrogate of muscle function from routine diagnostic work-up, which may be applied to a wide range of patient populations, both for baseline assessment as well as for monitoring during course of disease. It is unclear how CT-derived measurements of skeletal muscles change during course of disease, particularly in critically ill patients. Therefore, a narrow time window with regard to ECMO implantation needed to be selected for inclusion to this exploratory study. We acknowledge that this may have limited the number of includable patients. However, the results of this study indicate a strong association between CT-derived skeletal muscle measurements and outcome in ECMO patients and therefore warrant larger and particularly prospective studies, which may deepen and eventually confirm its findings. Within these studies, the role of FMF may be also be compared to other methods for skeletal muscle assessment as due to the study design and composition of the study population we were not able to directly compare FMF to functional and laboratory markers of low muscle quality. However, previous studies indicated that CT-based muscle quality assessment provides reliable estimates of muscle function^32^ and that particularly FMF is related to laboratory markers of frailty^21^. Finally, the salvage character of the therapy limited the number of includable patients. Therefore, our results should be considered exploratory and warrant larger and prospective studies which eventually may confirm its findings.

To conclude, we propose FMF as an objective, clinically applicable indicator of muscle quality, which is readily available from clinical CT and, applied as a baseline measure of muscle quality, provides prognostic information for long-term outcome in ECMO patients. Future studies may evaluate its role compared to clinical measurements of muscle function as well as its clinical value in other patient cohorts.

## Data Availability

The datasets generated during and/or analyzed during the current study are available from the corresponding author on reasonable request.
